# Identifying occupational health and safety risks among environmental health officers in Australia and New Zealand through an online survey

**DOI:** 10.1097/MD.0000000000033270

**Published:** 2023-03-24

**Authors:** Garry Dine, Sue Reed, Jacques Oosthuizen, Edmore Masaka

**Affiliations:** a School of Medical and Health Sciences, Edith Cowan University, Joondalup, Perth, WA, Australia.

**Keywords:** environmental health officers, occupational health and safety, workplace exposure

## Abstract

To identify the occupational health and safety (OHS) risks among environmental health officers (EHOs) in Australia and New Zealand. The objectives were to profile and compare OHS experiences from different countries and regions to gain a regional perspective on OHS hazards that impact EHOs.

An online hazard exposure survey was conducted among 339 EHOs (Australia: n = 301, 88.8%; New Zealand: n = 38, 11.2%). The Mann–Whitney *U* test was used to compare 2 ordinal data groups, the Kruskal–Wallis *H* test was used for more than 2 ordinal groups, and the independent samples *t* test was used to compare the means of 2 independent groups where the dependent variables were normally distributed. Multiple regression techniques were used to analyze workplace incidents and age groups. A high degree of similarity in the types of workplace exposures and risk perceptions as well as concerns with organizational OHS management commitment were observed among EHOs from the 2 countries. Workplace violence and physical and psychosocial demands were the most commonly reported OHS hazards. Employer type, sex, and age group were significantly related to workplace exposure and OHS experience among EHOs in both countries. This study provides a profile of workplace exposure in the environmental health profession in the 2 countries and offers recommendations for the implementation of preventive action.

## 1. Introduction

While every occupation is associated with potential exposure to hazards and risks of illnesses and injuries, little is known about the trends and magnitude of workplace health and safety risks among environmental health officers (EHOs). As in other professions, unsafe behavior and occupational health and safety (OHS) practices among EHOs can be motivated by internal and external factors.^[1]^ It is also suggested that EHOs perceive workplace risks as an inherent part of their jobs, which influences their risk perceptions and tolerance.

The objectives of this study were to determine if any differences in EHOs workplace health and safety experiences exist between Australia and New Zealand based on their existing practices of environmental health, and to determine whether employer description, years of work experience, and other demographic factors (i.e., age, sex, employer type, and years worked as an EHO) may be used to predict workplace exposures among EHOs.

### 1.1. Environmental health workforce

The environmental health workforce comprises generalist EHOs or specialist professionals who focus on particular aspects, such as environmental noise, public health engineering, and food hygiene. EHOs are entrusted with implementing measures to protect public health and are mostly employed by the local government, also known as councils. The approach to environmental health is similar in Australia and New Zealand; it focuses on assessing, correcting, and preventing the impact of environmental health stressors on communities. However, the work scope of EHOs in New Zealand is narrower, with a stronger focus on health compliance. In both countries, the functions of EHOs may vary slightly from council to council, depending on jurisdiction, environment, and resource levels. These functions include the investigation, sampling, measurement, and assessment of hazardous environmental agents in various environmental media and settings. This informs the formulation of appropriate recommendations for the implementation of protective interventions to control health hazards. Furthermore, EHOs develop, promote, and enforce guidelines, policies, and laws related to environmental health, and they engage with community members to understand, address, and resolve environmental health risks. EHOs are also involved in the review of land use and building approval. ^[2,3]^

In Australia and New Zealand, the environmental health sector is a relatively small workforce that performs a fundamental function in the state and local governments. The professional cohort is estimated to comprise approximately 3600 and 300 full-time-equivalent employees in Australia and New Zealand, respectively. There is currently no available literature that describes the impact of OHS risk on EHOs existing work practices in Australia or New Zealand. Anecdotal evidence suggests that there is a high degree of similarity in OHS experiences amongst EHO workforces in both countries as they share similar environmental health legislative tools, standards, and approaches. Furthermore, the work setting and type of environmental health services available are more or less the same.

### 1.2. Working conditions

EHOs work in challenging and hazardous environments. These roles are associated with a substantial amount of fieldwork and travel. Depending on the level and type of environmental health services provided, many EHOs work long and irregular hours. They may be exposed to similar physical, chemical, biological, and psychological hazards as workers from industries such as agriculture, manufacturing, construction, and law enforcement, as well as public servants. However, 1 significant workplace hazard affecting EHOs is workplace violence. ^[4,5]^ As part of their regulatory function, EHOs are involved in a variety of environmental health compliance tasks such as regulatory inspections, complaint investigations, and surveillance work. These tasks place them in direct contact with dissenting business owners, offenders, and irate individuals. In some situations, it is necessary for EHOs to use their “power of entry” to access private premises in cases of serious potential public health concerns. They are occasionally denied entry, and these obstructions are often accompanied by aggressive and abusive behavior. As multi-skilled and highly trained professionals, EHOs are often deployed during public health emergencies. An example is the corona virus disease 2019 (COVID-19) pandemic, in which EHOs were required to enforce public health directions and protocols. In other cases, EHOs support the community in recovering from natural disasters, including cyclones, floods, and bushfires. These situations expose them to potential hazards, and little is known about preventive measures taken to ensure their safety. EHOs are frequently the first responders to incidents, exposing them to hazardous materials, such as asbestos.^[6]^ Other documented workplace hazards include workplace and body stress, as well as vehicular accidents.^[7]^ In this study, we outline the main workplace exposures impacting EHOs and provide information related to their diverse, often-misunderstood roles.

## 2. Materials and Methods

The Australian National Hazard Exposure Worker Surveillance Survey (NHEWS) was adapted for this study.^[8]^ This instrument was developed by the Australian Safety and Compensation Council in 2008. The original NHEWS instrument considered some areas that were not directly relevant to EHOs or the research question. The survey was adapted to our research context by deleting some questions related to tasks, behaviors, or other areas not relevant to EHOs. Further, several questions were added to cover areas not specifically addressed in the NHEWS instrument, such as asbestos exposure, COVID-19, and workplace incidents. The survey comprised nominal, ordinal, Likert scale, and ratio scale questions. The amended survey contained 34 questions, some of which had multiple parts and were structured around 8 key themes (Table 1). The survey used “skip logic,” which sent respondents to a future point in the survey based on how they answered a question, and it was designed to be completed in under 20 minutes. The survey was piloted with a convenience sample of EHOs (n = 20) using the Qualtrics online platform. The identified issues and concerns were addressed before the dissemination of the main survey, which was deployed in June 2021.

**Table 1:** Questionnaire themes, data items, and concepts.

Themes	Data items and concepts
Demographics	Age, sex, level of education, employment position, employer description, and duration of employment
Working arrangements	Work hours and work schedule
Physical hazards	Sun sensitizers, noise, dust, physical demand, working position, and posture
Chemical hazards	Fumes, vapors, and hazardous substances
Biological hazards	Infectious materials
Psychosocial hazards	Job demand, workload, supervisor and work colleagues support, decision authority, job security, and required skill
Work symptoms	Fatigue, musculoskeletal discomfort/pain, and stressfulness of work
Workplace violence	Verbal and physical violence, threat of violence, and training to deal with workplace violence
Workplace asbestos exposure	Asbestos exposure and knowledge
COVID-19	Exposure and knowledge
Incident reporting and workers claim	Incident reporting, mechanism of injury, and workers compensation claim

A purposive sampling approach was used for the cross-sectional survey, allowing any potential respondent to self-select into the sample and thus avoid potential sample bias. This sample approach was considered appropriate as EHOs are a small workforce (Australia: n = 3600; New Zealand: n = 300), the population is well defined, and there are limited primary data sources on the subject matter. The survey was distributed via professional environmental health associations within the 2 respective countries (Environmental Health Australia and New Zealand Institute of Environmental Health). Both professional associations nationally represent EHOs. Participants were contacted via email by their professional environmental health association with a covering information letter from the researchers and an embedded link to allow direct connection to the survey form. The survey was conducted for 3 weeks, and a follow up email was sent at the end of the second week.

Descriptive statistics were used to provide information about the variables in the datasets and identify the relationships among them. Nonparametric tests (Kruskal–Wallis *H* test and Mann–Whitney *U* test) were used to determine statistical significance between 2 or more variables that were not normally distributed. An independent *t* test for normally distributed continuous data was used to compare the occurrence of workplace violence between the sexes. Multiple regression modeling was used to analyze the relationship of occurrence of workplace incidents with “age group” and the “number of years worked as an EHO.”

All participants signed a consent form and anonymity and confidentiality were ensured. This study was approved by the Edith Cowan University Human Research Ethics Committee (REMS NO: 2020-01757-DINE).

## 3. Results

### 3.1. Sample

A total of 339 EHOs from Australia (n = 301; 88.8%) and New Zealand (n = 38; 11.2%) participated in this survey. Incomplete surveys were not included. In Australia, there were 155 (51.5%) male and 145 (48.2%) female participants, while New Zealand had 14 (36.8%) male and 24 (63.2%) female participants. Most participants were employed by local governments [metropolitan local government (36.9%), regional local government (38.1%), and rural local government (16.8%)]. The average lengths of environmental health work experience for Australian participants were 17.34 years for male and 13.26 years for female participants; in New Zealand, they were 20.43 years for male and 9.5 years for female participants. Most participants (Australia 90.7% and New Zealand 94.7%) had a bachelor’s degree or higher in a related environmental health or environmental science field.

### 3.2. Organizational OHS management commitment

There were some significant differences across employer description categories in Australia (i.e., metropolitan local government, rural local government, regional local government, and state health department) in response to questions about general OHS concerns. A Kruskal–Wallis test showed a significant difference in the responses of EHOs in different employer categories to the question on whether they “normally have sufficient time to take appropriate safety precautions while completing their work-related duties” (H [5] = 12.75, *P* = .03). post hoc pairwise comparisons further showed that regional local governments were significantly different from metropolitan local governments (*P* = .03), and regional local governments were significantly different from rural local governments (*P* = .02). Participants employed by a metropolitan local government were more likely to have sufficient time to take appropriate safety precautions while completing their duties than participants employed by a regional or rural local government.

Regarding “appropriate personal protective equipment being supplied and readily available,” a Kruskal–Wallis test showed a significant difference among these employer categories (H [5] = 21.70, *P* = <.001). post hoc pairwise comparisons showed that the state health department was significantly different from rural local governments (*P* = .04), regional local governments were significantly different from rural local governments (*P* = .007), and metropolitan local governments were significantly different from rural local governments (*P* = .02). Participants from a rural local government were less likely to have appropriate personal protective equipment being supplied and readily available compared to those from the state health department and metropolitan and regional local governments.

There was a significant difference across the employer categories in relation to the following statement: “It’s easy for me to manage my work duties with family responsibilities” (H [5] = 11.81, *P* = .04). Post hoc pairwise comparisons showed that regional local governments were significantly different from the state health department (*P* = .05). It appears easier for EHOs employed by the state health department to manage work duties with family responsibilities compared to those from a regional local government.

For the question on whether the participants’ “work area is adequately staffed,” a significant difference across the employer categories was demonstrated (H [5] = 13.64, *P* = .02). post hoc pairwise comparisons showed that metropolitan local governments were significantly different from rural local governments (*P* = .01) and the state health department (*P* = .006), and regional local governments were significantly different from the state health department (*P* = .03). EHOs from a rural local government and the state health department were more likely to disagree that their work area was adequately staffed.

No significant difference was observed across employer categories in the New Zealand dataset.

### 3.3. Psychosocial demands

To assess psychological work demands, participants were asked questions related to time and cognitive demands, and job satisfaction and control. Most EHOs indicated that they found their job moderately stressful (64.9%), while others perceived it as extremely (4.4%) or mildly stressful (30.7%). There was no significant difference in the indicated levels of job stress across the demographic variables for either country.

Mann–Whitney *U* tests showed a significant sex difference among the Australian respondents for the following statements: “My fellow workers respect me” (*U* = 9910.5, *P* = .04); “My fellow workers are willing to listen to my work-related problems” (*U* = 9942.5, *P =* .05); and “I have some say over the way I work” (*U* = 9876.5, *P* = .03). Male participants were more likely to be in strong agreement with these statements than their female counterparts.

There was a significant difference across the age group categories among New Zealand participants for the statement, “I have some say over the way I work” (H [5] = 12.49, *P* = .03). post hoc pairwise comparisons showed that the 55 to 64 age group was significantly different from the 18 to 24 group (*P* = .02) and the 25 to 34 group (*P* = .02). Older participants were more likely to agree with the statement than younger participants.

### 3.4. Work-related musculoskeletal pain and fatigue

**Figure 1.**
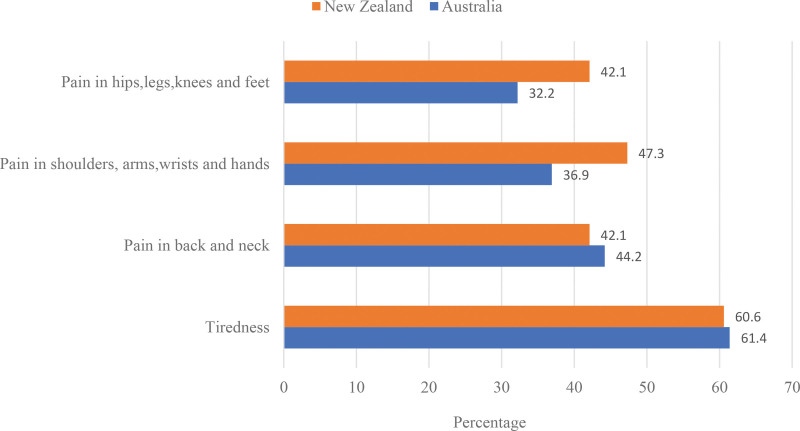
Percentage of environmental health officers who reported experiencing work-related musculoskeletal pain and fatigue as a result of the physical demands of their job the week before they completed the survey (Australia n = 301; New Zealand [NZ] n = 38).

Figure 1 shows the percentage of EHOs who reported that they experienced work-related musculoskeletal pain and fatigue because of the physical demands of their job the week before they completed the survey.

Mann–Whitney *U* tests showed a significant difference in participants in Australia who reported experiencing pain in the neck or back (*U* = 13,055.5, *P* = .01); male participants were less likely to experience neck or back pain. Significant differences across sex were also observed in shoulder or arm pain among Australian participants (*U* = 12,909.0, *P* = .02); male participants were less likely to experience shoulder or arm pain. A Kruskal–Wallis test showed that there was a significant difference across age groups for pain in the hips, legs, knees, or feet (H [5] = 13.87, *P* = .020). post hoc pairwise comparisons showed that the 18 to 24 age group was significantly different from the 65 to 74 group (*P* = .02); the 55 to 64 age group was significantly different from the 25 to 34 group (*P* = .04); and the 45 to 54 age group was significantly different from the 25 to 34 (*P* = .02) and 65 to 74 (*P* = .01) groups. Participants in the older age groups were more likely to experience pain in the hips, legs, knees, or feet. There were no significant differences across other demographic variables (i.e., level of education, employer description, and position description) for work-related musculoskeletal pain and fatigue among Australian respondents.

A Mann–Whitney *U* test showed a significant difference across sex for New Zealand participants who experienced fatigue the week before they completed the survey (*U* = 240.0, *P* = .03), with male participants less likely to report fatigue. There were no significant differences across the other demographic variables for musculoskeletal pain and fatigue among the New Zealand participants.

### 3.5. Workplace violence

Respondents were asked to report their experiences of different types of workplace violence in the past 12 months. Verbal violence (i.e., being shouted or sworn at, called names, or verbally confronted) was the most common type of violence reported by the EHOs [*M* = 3.61 (Australia) and 2.79 (New Zealand)]. The number of times participants had witnessed another EHO being subjected to physical violence (i.e., being hit, kicked, grabbed, shoved, bitten, or having an object thrown at them) was the following on average: *M* = 1.12 (Australia) and 1.05 (New Zealand).

An independent samples t-test was conducted among Australian participants to compare the “occurrence of being threatened with physical violence” between male and female participants. There was a significant difference (*t* [279.2] = 2.41, *P* = .02) between male (*M* = 1.59, *SD* = 1.092) and female (*M* = 1.32, *SD =* .781) participants regarding the number of times they had been threatened with physical violence. The magnitude of the difference in the means (mean difference = .263, 95% confidence interval:.048–.478) was significant.

A significant difference was also observed across age groups in Australia for being “threatened with physical violence” (H [5] = 11.60, *P* = .04). post hoc pairwise comparisons showed that the 18 to 24 age group was significantly different from the 65 to 74 group (*P* = .02), and the 25 to 34 age group was significantly different from the 65 to 74 group (*P* = .005). The prevalence of being “threatened with physical violence” was higher among older EHOs.

Although male participants were more likely to report violent incidents, there was no significant difference in the occurrence of workplace violence across demographic variables among the New Zealand participants.

### 3.6. Reported workplace incidents by mechanism of the incidents

**Figure 2.**
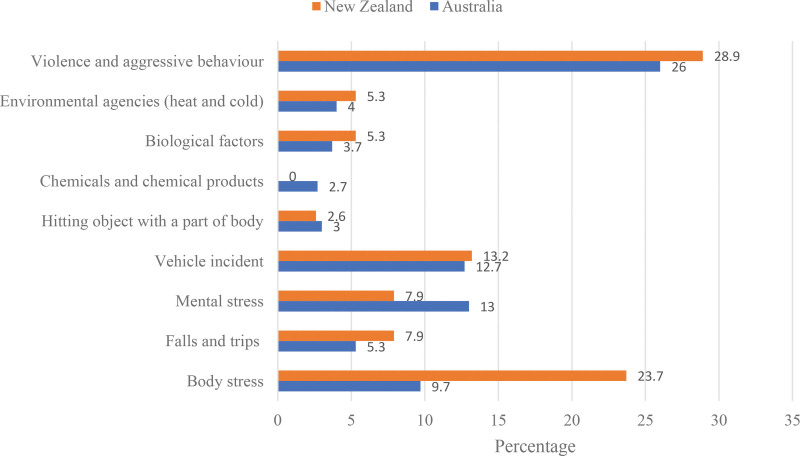
Reported workplace incidents by mechanism for environmental health officers in Australia and New Zealand (NZ). Participants were asked to specify the number of workplace incidents and their mechanisms that they reported to their employers in the 12 months preceding the survey (Australia: n = 301; NZ: n = 38).

Participants were asked to specify the number of workplace incidents and their mechanisms that they formally reported to their employer in the 12 months preceding the survey. Workplace violence and aggressive behavior were the most common types of incidents reported, followed by mental stress, vehicular incidents, and body stress (Fig. 2). The mean number of reported incidents was 2.12 and 2.47 for Australia and New Zealand, respectively. The mean ranks across sex were as follows: Australia: male = 151.27, female = 149.67 and New Zealand: male = 22.11, female = 17.98.

Younger participants were less likely to report workplace incidents than older participants [age group mean: Australia = 18–24 (1.83), 25–34 (1.87), 35–44 (2.08), and 45–54 (2.05); New Zealand = 18–24 (1.00), 25–34 (2.86), 35–44 (3.30), and 45–54 (2.31).

A multiple linear regression analysis using the enter method was conducted to assess whether age group and number of years working as an EHO significantly predicted workplace incidents among Australian participants. The model was not significant [*R^2^* =.007, *F* (2, 298) = 1.004, *P* = .37]. The regression analysis results suggest that age group and number of years working as an EHO explained only 0.7% of the variance. There were no significant differences across demographic variables and the number of reported workplace incidents in either country.

## 4. Discussion

### 4.1. Organizational OHS management commitment

There were significant differences in some general OHS concerns across employer types among Australian participants. Participants who worked for a metropolitan local government and state health department benefited from better OHS organizational commitment than their colleagues who worked in smaller regional and rural settings. Some of the key factors affecting OHS in small and remote organizations are low management and training skills, lack of resources, and burden of compliance.^[9]^ In Australia, while a few local governments have or are working toward accredited OHS management systems, there is a view that most local governments have been reactive in dealing with OHS performance, especially smaller local governments.^[10]^ The management commitment of OHS in most local governments in Australia is suggested to be basic, despite the presence of written policies and good intentions.^[11]^ Occasional health and safety arrangements in Australia and New Zealand have many common features,^[12,13]^ and the situation of OHS in local governments in New Zealand is likely to be the same, especially among smaller local governments.

Concerns related to insufficient time to take appropriate precautions while completing a task, availability of appropriate personal protective equipment, work–life balance, and workload were more likely to be reported by EHOs from regional and rural local governments. Similar observations have been made among other health professionals in Australia who work in remote and regional areas compared to those who operate in metropolitan settings.^[14]^ This phenomenon has been associated with poor safety culture, isolation, resources, a safe environment, and appropriate training.^[15]^ EHOs can be highly responsive; they are often required to urgently attend to hazardous environmental health emergencies, such as sewage spills, events related to uncontained fibrous asbestos, or toxic chemical spills. It is vital that EHOs have access to appropriate personal protective equipment, adequate time to assess personal safety, and a precautionary approach. Organizations employing EHOs must ensure that clear OHS processes relating to different environmental health emergencies are developed, together with the provision of appropriate protective equipment and training.

The finding that more EHOs from rural and regional local governments indicated that their work areas were inadequately staffed is not surprising. Turnover rates in rural and regional local governments have been reported to be >20%,^[16]^ the reasons for which include lack of career progression in smaller councils, inability of councils to compete with higher remuneration packages in cities, and the lack of stability and leadership in senior management.^[16]^ EHOs in these settings have to take on additional duties that are normally performed by town planners and building surveyors in larger local governments. This may contribute to additional work stress and thus impact EHOs work–life balance.

Employees turnover intention is heavily influenced by the commitment of organizations to ensuring a safe workplace.^[17]^ Management leadership is central to the implementation and supervision of OHS to reduce turnover intention in organizations.^[18]^ Regional and rural local governments should prioritize appropriate OHS strategies to address employees workplace concerns and provide ongoing resources to sustain OHS standards.

### 4.2. Psychosocial demands

The study indicates that there are potential workplace relationship issues and a sense of being undervalued experienced by EHOs in both Australia and New Zealand. These sentiments are expressed more by female EHOs; however, it was beyond this study’s scope to investigate factors that are potentially responsible for these views. The feeling of being unseen and unheard is a common theme among the EHO workforce, and there is a plea by public health scholars for this oversight to be addressed.^[19]^ Many employees deserve far more respect at work than they receive^[20]^; this is certainly true for EHOs, considering their efforts and contributions to the community as frontline public health professionals. Rogers and Ashforth^[20]^ discussed 2 distinct types of respect at work: “generalized respect” is the sense that “we” are all valued in this organization, and “particularized respect” is the sense that the organization values “me” for particular attributes, behaviors, and achievements. It has been suggested that EHOs are deprived of both types.^[19]^ In Australia, the work of EHOs is not well understood or supported by local governments, as it is not part of the local governments broader strategic framework.^[21]^ Anecdotal evidence suggests that there are growing concerns about low morale and a lack of contentment within the EHO workforce in both countries.

Participants also indicated that they were not listened to and had limited say in the way they worked. There were significant differences across sexes for this finding, which was expressed mostly by female and younger participants. It is not clear from this study whether this is a problem within the environmental health leadership framework or a broader management issue experienced by EHOs within their organization. Given that these concerns have been reported more often by female and younger EHOs, this may also raise concerns about the power dynamics at play. It has been argued that the balance of power has been shifting toward employers in recent years.^[22]^ Further research is needed to verify whether there is indeed an issue of power dynamics affecting EHOs. Organizations that employ EHOs need to recognize the benefit of high-quality relationships between leaders and their staff to ensure optimal performance and worker satisfaction.^[23]^

### 4.3. Work-related musculoskeletal pain and fatigue

There was a significant difference in participants in Australia who reported experiencing work-related musculoskeletal pain across the categories of age and sex. Work-related musculoskeletal problems are common among different work groups, and studies have shown a higher prevalence among female ^[24–26]^ and older ^[27–29]^ workers.

EHOs must spend a significant period of time in the field, often on difficult terrain, to investigate environmental health issues. Some tasks (e.g., sample collection, inspection of confined spaces, lifting objects) require repetitive movements of the upper and lower body. It is possible that the prevalence of musculoskeletal pain among EHOs is related to the nature of their tasks and the challenging environment in which they are performed. There is evidence to link musculoskeletal problems with fatigue ^[30–32]^ and unreasonable workloads.^[33,34]^ A probable hypothesis is that unreasonable workload, musculoskeletal pain, and experience of fatigue are interlinked workplace issues within EHOs profession, and a holistic approach is necessary to reduce or eliminate them. The preventive approach must consider the different segments of the environmental health workforce and address both psychosocial and physical work demands.

### 4.4. Workplace violence

Data from this study indicated that workplace violence is a significant hazard for EHOs. Compared to other occupational settings, workplace violence manifests differently among EHOs. In Australia, some studies in the late 1990s worked to define what is meant by workplace violence.^[35,36]^ The category of violence that commonly impacts EHOs is termed “occupational violence,” which occurs when the job is performed and perpetrated by outside parties such as customers and clients.^[37]^ This is different from intraorganizational violence, in which the conflict is between workers. The risk factors for workplace violence among EHOs include inspections or enforcement duties. EHOs require a high frequency of interpersonal contact with customers, including business owners, developers, builders, and property owners. EHOs often adopt a stringent and sometimes legislative approach when dealing with these individuals, as their action or inaction often results in health risks or negative impacts on the environment. This enforcement-centric work characteristic is associated with public-initiated violence.^[37]^

EHOs are occasionally obstructed from doing their jobs, and these obstructions are often accompanied by aggressive and abusive behavior.^[38]^ During the COVID-19 pandemic, there have been numerous media headlines of EHOs receiving violent treatment or being subjected to violence in their efforts to implement regulatory measures to combat the pandemic.^[39,40]^ Violence against local government officers in Australia and New Zealand has been reported more frequently in the media in recent years.^[41,42]^ In July 2019, local councils across Victoria, Australia called for an increase in sentencing laws owing to a rise in assaults on local government authorized officers.^[41]^

Age and sex differences in workplace violence are not unique to the EHO workforce. Multiple studies from other health workforces have found a higher prevalence of workplace violence among male workers.^[43–45]^ The association of workplace violence with older age groups has been shown among nurses in Australia.^[44]^ A recent study from the United States also suggested that older workers experience more severe episodes of physical assault.^[46]^ It is necessary to further investigate the distinct differences in workplace violence across the different subsets of EHOs, as these are not yet well-described, and more details about the types of incidents would allow for targeted prevention strategies.

### 4.5. Reported workplace incidents by mechanism

The most common mechanisms of workplace incidents reported in this study were workplace violence, vehicular accidents, and body and mental stress. This trend is similar to what has been previously documented among EHOs in Australia^[7,47]^ and the trend in the general Australian^[48]^ and New Zealand^[49]^ workforce.

In a study from Western Australia, EHOs perceived themselves as being at a risk of exposure to workplace stress and violence, injury from sharp objects, and slips, trips, and falls. Similarly, results from a retrospective analysis of workplace incident data among EHOs, collected from local governments in Australia, showed that the most common mechanisms of workplace incidents were vehicular accidents (28%), followed by mental stress (16%) and slips, trips, and falls (15.3%).^[47]^

No significant associations were found between the reporting of workplace incidents and demographic variables. However, the prevalence of workplace incidents was higher among male and older participants. Key work health and safety statistics for Australia in 2021 showed that the incidence rate for serious workers compensation claims per 1000 employees by sex was 12 in male workers as compared to 7.6 among female workers; incidence rates were higher in the older age groups.^[48]^ New Zealand injury statistics for 2020 showed that men were responsible for 69% of all claims compared to women (31%), with the highest incidence among older workers.^[50]^

Similar workplace incident prevention strategies used in other sectors can be considered for the EHO workforce. These include de-escalation techniques, management of assaultive behavior, and training that focuses on behaviors that could result in violence.^[51]^ The government’s legislation to protect EHOs from violence must also be enacted.^[52]^ Workplace exercise and risk analysis for early identification of musculoskeletal injuries have been found to be beneficial.^[53]^

The key objectives of this study were to profile and compare OHS experiences from different countries and regions to gain a regional perspective on OHS hazards that impact EHOs. A high degree of similarity in the types of workplace exposures and risk perceptions, and concerns with organizational OHS management commitment, was observed among EHOs from both countries. Experience of workplace violence and issues associated with physical and mental work demands were the most common OHS hazards reported by the EHOs. There were significant associations between demographic factors (i.e., age group, sex, employment description, location, and level of education) and workplace psychosocial demands and violence, risk perceptions, and work symptoms (musculoskeletal pain, fatigue). Based on the sample method used and the fact that the EHO workforce is relatively small, the findings can be extended to the general EHO workforce.

The findings deepen the current understanding of the function of environmental health workforce and the types of workplace hazards that may impact EHOs. The study provides important evidence that can be used to inform policy and preventive OHS strategies within the environmental health workforce. Furthermore, the results provide preliminary OHS information about the environmental health workforce that can be used for subsequent research.

### 4.6. Limitations

We relied on EHOs self-reported exposure to hazards and symptoms; therefore, respondents with biases may have selected themselves into the sample. We also did not address the causes of OHS hazards among EHOs. Furthermore, the survey was conducted during the COVID-19 pandemic, and EHOs participation and responses may have been influenced by related circumstances.

## 5. Conclusions

This study provides a profile of workplace exposure for EHOs in Australia and New Zealand and is sufficient for informing preventive action. Similarity was observed in the types of workplace exposure, risk perceptions, and general OHS concerns among EHOs from both countries. There were significant associations between demographic factors (i.e., age group, sex, and employer description) and workplace exposure, risk perceptions, and work symptoms. Workplace violence and physical and psychosocial demands were the main OHS hazards reported by the EHOs.

## Acknowledgments

The authors wish to thank Environmental Health Australia and the New Zealand Institute of Environmental Health for their collaboration in this study. A big thank you for all the EHOs who participated in the survey.

## Author contributions

**Conceptualization:** Garry Dine, Sue Reed, Jacques Oosthuizen.

**Formal analysis:** Garry Dine.

**Investigation:** Garry Dine.

**Methodology:** Garry Dine.

**Supervision:** Sue Reed, Jacques Oosthuizen, Edmore Masaka.

**Validation:** Jacques Oosthuizen.

**Writing – original draft:** Garry Dine.

**Writing – review & editing:** Sue Reed, Jacques Oosthuizen, Edmore Masaka.
